# A Δ-learning strategy for interpretation of spectroscopic observables

**DOI:** 10.1063/4.0000215

**Published:** 2023-11-06

**Authors:** Luke Watson, Thomas Pope, Raphael M. Jay, Ambar Banerjee, Philippe Wernet, Thomas J. Penfold

**Affiliations:** 1 Chemistry, School of Natural and Environmental Sciences, Newcastle University, Newcastle upon Tyne NE1 7RU, United Kingdom; 2 Department of Physics and Astronomy, Uppsala University, 751 20 Uppsala, Sweden

## Abstract

Accurate computations of experimental observables are essential for interpreting the high information content held within x-ray spectra. However, for complicated systems this can be difficult, a challenge compounded when dynamics becomes important owing to the large number of calculations required to capture the time-evolving observable. While machine learning architectures have been shown to represent a promising approach for rapidly predicting spectral lineshapes, achieving simultaneously accurate and sufficiently comprehensive training data is challenging. Herein, we introduce Δ-learning for x-ray spectroscopy. Instead of directly learning the structure-spectrum relationship, the Δ-model learns the structure dependent difference between a higher and lower level of theory. Consequently, once developed these models can be used to translate spectral shapes obtained from lower levels of theory to mimic those corresponding to higher levels of theory. Ultimately, this achieves accurate simulations with a much reduced computational burden as only the lower level of theory is computed, while the model can instantaneously transform this to a spectrum equivalent to a higher level of theory. Our present model, demonstrated herein, learns the difference between TDDFT(BLYP) and TDDFT(B3LYP) spectra. Its effectiveness is illustrated using simulations of Rh L_3_-edge spectra tracking the C–H activation of octane by a cyclopentadienyl rhodium carbonyl complex.

## I. INTRODUCTION

Driven by the rapid progress in high-brilliance third- and fourth-generation light sources such as synchrotrons and x-ray free-electron lasers (XFELs), the past decade has witnessed significant advances in the theory of core-hole spectroscopies.^1^ However, although computational analysis of experimental observerables is increasingly commonplace, for complex systems an accurate interpretation can be challenging, calling for computationally expensive high-level quantum chemistry methods.^2–5^ This challenge is compounded during the analysis of time-resolved experiments^6,7^ where a larger number of calculations need to be performed to capture the time-evolution of the observable.^8–11^

Supervised machine-learning/deep learning algorithms,^12^ i.e*.,* multilayer models aimed at extracting and learning patterns represented in data, have emerged as a potential approach for overcoming this challenge. Recently deep neural networks (DNN) capable of predicting the line shape of x-ray absorption (XAS)^13–20^ and emission (XES)^21,22^ spectra have been developed. The key to any machine learning model is the quality of the data with which it is trained. To achieve accurate DNNs capable of converting input structures into spectral lineshapes, in a manner akin to quantum chemistry calculations, two distinct approaches for generating training data have been explored. The first approach, referred to as “Type I”, focuses on achieving generality in the sense that it is able to simulate an XAS/XES spectrum for an arbitrary absorbing atom in any coordination environment for a given absorption edge. On the other hand, the second approach, “Type II”, is tailored to a specific problem and is therefore trained using data for a single class of systems.^23–26^

A general Type I model is preferable, as it avoids the time-consuming requirement to develop a new model for each specific problem. However, the main challenge associated with developing accurate and generalizable training sets for prediction of x-ray absorption near-edge structure (XANES) spectra is scale. Indeed, recent DNN models for predicting XAS spectral lineshapes of transition metal K-edges^16^ have been trained using molecules from the tmQM training set^27^ containing a single geometry of the mono-metallic complexes harvested from the Cambridge structural database (CSD).^28^ While this has been shown to be accurate when used to predict spectral shapes of compounds in a similar chemical space, large uncertainties arise when considering complexes with multiple absorbing atoms or a strongly distorted from their equilibrium geometry.^15,29^ Ultimately achieving comprehensive coverage of the chemical space is a significant challenge, especially when seeking to develop a training set using a high-level theory with large computational burden for each sample.

**FIG. 1.**
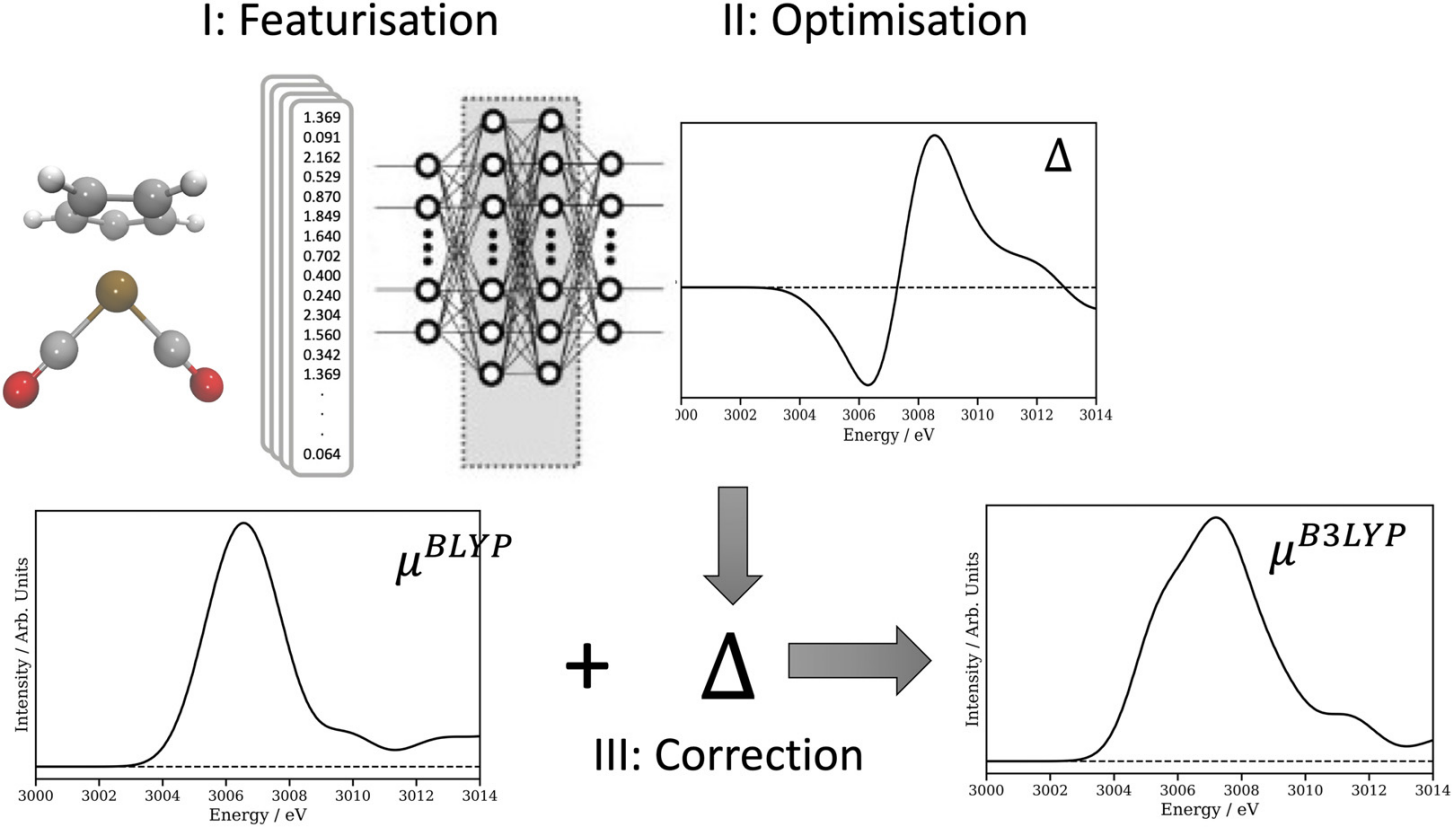
Schematic of the Δ-learning approach adopted in this work. The featurized local geometries around the Rh complexes used in the training set (I) are inputs, while the difference between their TDDFT(BLYP) and TDDFT(B3LYP) calculated Rh L_3_-edge XANES spectra are outputs (II). Once optimized, the predicted difference is added the TDDFT(BLYP) spectrum to recreate a spectrum equivalent to TDDFT(B3LYP).

One approach to overcome this is to use a composite strategy, Δ-learning, as introduced by Ramakrishnan *et al.*^30^ The concept behind this is to use the machine-learning models to correct the properties obtained from a computationally inexpensive approximate quantum calculation to those corresponding to a higher-level, but ultimately more computationally expensive approach. Importantly, this approach has been demonstrated to exhibit a measurable advantage for small and selected training sets^30^ and the success of this strategy has led to a number of successful applications used across quantum chemistry.^31–33^ In the present work, shown schematically in Fig. 1, we implement and deploy a Δ-learning strategy for simulating x-ray spectra, i.e*.,*

$$\mu {\left(E\right)}^{H}=\mu {\left(E\right)}^{L}+\Delta {\left(E\right)}^{ML},$$ (1)

where $\mu {\left(E\right)}^{H}$ is the spectrum calculated at a high level of theory, $\mu {\left(E\right)}^{L}$ is the spectrum computed at the lower level of theory and $\Delta {\left(E\right)}^{ML}$ is the correction learnt by our DNN. It is noted that this approach bears some resemblance to the spectral warping approach of Prentice and Mostofi^34^ who applied a series of linear transformations to the semi-local TDDFT spectrum, in order to obtain a good estimate of the hybrid TDDFT spectrum. Our results, which are inherently non-linear due to the use of the DNN, applied to the Rh L_3_-edge, demonstrates that the Δ-learning strategy can quickly learn the difference between TDDFT(BLYP) and TDDFT(B3LYP) computed spectra, providing an composite method for obtaining accurate core-hole spectra at reduced computational cost, as $\mu {\left(E\right)}^{H}$ can be achieved using $\mu {\left(E\right)}^{L}$ and the predicted $\Delta {\left(E\right)}^{ML}$ from the developed model. The accuracy of this approach is further exemplified by simulating Rh L_3_-edge spectra tracking the C–H activation of octane by a cyclopentadienyl rhodium carbonyl complex.^35^ This system has received significant interest as a model complex for transformation of saturated hydrocarbons through C–H bond activation.^36,37^ Recently, Jay *et al.*^35^ used time-resolved x-ray spectroscopy to track the charge-transfer interactions during C–H activation and revealed changes in oxidation state as well as valence-orbital energies and character from femtosecond Rh-alkane bond formation to nanosecond C–H bond cleavage. In the present work, we use our Δ-learning model to demonstrate that it can accurately reproduce the TDDFT(B3LYP) spectra from a TDDFT(BLYP) starting point, with the resulting spectra closely resembling the experimental results.

## II. METHODS AND COMPUTATIONAL DETAILS

### A. Training data and quantum chemistry simulations

Our reference datasets comprise of 1124 x-ray absorption site geometries of Rhodium complexes harvested from the transition metal Quantum Machine (tmQM) dataset.^27,28^ This dataset was extracted from the 2020 release of the Cambridge Structural Database (CSD) and subsequently optimized at the GFN2-xTB level of theory. Full details of the construction and composition of the tmQM dataset can be found in Ref. 27.

The Rh L_3_-edge spectra for all of the structures in our reference datasets were calculated using a Restricted Excitation Window Time-Dependent Density Functional Theory (REW-TDDFT)^38^ as implemented in the ORCA quantum chemistry package.^39^ All spectra were computed twice using the BLYP and B3LYP^40–43^ exchange and correlation density functionals, with the difference between the two simulations used for training. It is noted that the choice of functional will systematically influence the absolute transition energies calculations^44^ and therefore before taking the difference, all the spectra calculated using BLYP and B3LYP were shifted by +19.5 and −5.5 eV respectively to match the absolute energy of the experimental white line. While this constant spectral shift applied to the whole training set could be a limitation to other types of spectroscopy, in the present case of x-ray spectroscopy, because the transitions derive from core orbitals, which are not involved in bonding and remain largely unchanged for different molecules, this approach ensure consistency for each sample. Scalar relativistic effects were described using a Douglas–Kroll–Hess (DKH) Hamiltonian of 2nd order.^45^ In all calculations an aug-cc-pVTZ-DK basis set was used for the Rh and all other elements used a DKH-def2-SVP basis set.^46,47^ The light–matter interaction was described using the electric dipole, magnetic dipole, and electric quadrupole transition moments.^44^ After calculation, each spectrum was broadened using a Gaussian function with a fixed width of 1.5 eV in all cases.

**FIG. 2.**
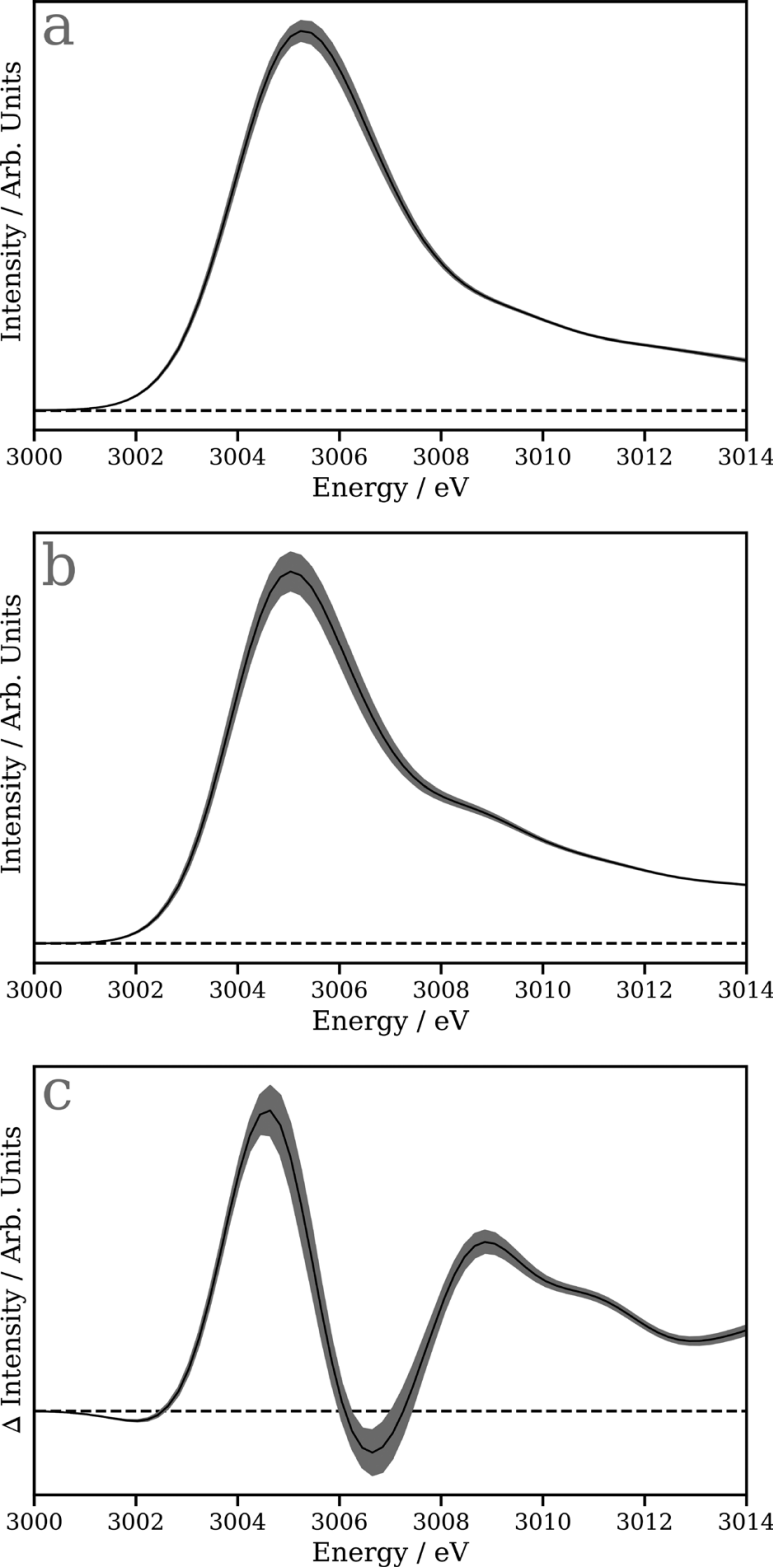
Mean (solid black line) and standard deviation ( ±σ; gray shaded region) of the 1124 Rh L_3_ x-ray absorption spectra used in the training set calculated using TDDFT(BLYP) (a) and TDDFT(B3LYP) (b). (c) Mean (solid black line) and standard deviation ( ±σ; gray shaded region) of the Δ between the TDDFT(BLYP) and TDDFT(B3LYP) spectra. The dashed line represents zero intensity.

Figure 2 shows the mean and standard deviation of the spectra within the training set calculated using TDDFT(BLYP) (a) and TDDFT(B3LYP) (b), while Fig. 2(c) shows the average and standard deviation of the Δ, i.e*.,* $\mu {\left(E\right)}^{B3LYP}-\mu {\left(E\right)}^{\mathrm{BLYP}}$. The mean difference shows a distinct derivative profile, indicating that the TDDFT(B3LYP) is generally shifted toward slightly lower energy. The positive feature at ∼3009 eV is associated with more pronounced features seen above the white line, as observed in Fig. 2(b).

### B. Network details and training

Our deep neural network (DNN) is based upon the multi-layer perceptron (MLP) model and closely follows that presented in Ref. 16. Briefly, the model comprises an input layer, two hidden layers, and an output layer. All layers are dense, i.e*.,* fully connected, and each hidden layer performs a nonlinear transformation using the hyperbolic tangent (*tanh*) activation function. The input layer contains the feature vector encoding the local environment around the absorbing atom performed via dimensionality reduction using the wACSF descriptor of Gastegger *et al.*^48^ Throughout this article, the input layer contains 49 neurons comprising a global (*G*^1^) function, 16 radial (*G*^2^) functions, and 32 angular (*G*^4^) functions.

Both hidden layers contains 256 neurons and the output layer comprises 250 neurons from which either the discretized Rh L_3_ spectrum or the discretized Δ, i.e*.,* $\mu {\left(E\right)}^{B3LYP}-\mu {\left(E\right)}^{\mathrm{BLYP}}$ is retrieved after regression. The internal weights, **W**, are optimized via iterative feed-forward and backpropagation cycles to minimize the empirical loss, J(W), defined here as the mean-squared error (MSE). Gradients of the empirical loss with respect to the internal weights, δJ(W)/δW, were estimated over minibatches of 32 samples and updated iteratively according to the Adaptive Moment Estimation (ADAM)^49^ algorithm. The learning rate for the ADAM algorithm was set to $1\times 10^{-4}$. The internal weights were initially set according to the He *et al.*^50^ uniform distribution. Unless explicitly stated in this article, optimization was carried out over 240 iterative cycles through the network commonly termed *epochs*. Regularization was implemented to minimize the propensity of overfitting; batch standardization and dropout were applied at each hidden layer. The probability, *p*, of dropout was set to 0.15, unless otherwise stated.

The XANESNET DNN is programmed in Python 3 with the TensorFlow^51^/Keras^52^ API and integrated into a Scikit-Learn^53^ (*sklearn*) data pre- and post-processing pipeline via the KerasRegressor wrapper for Scikit-Learn. The Atomic Simulation Environment^54^ (*ase*) API is used to handle and manipulate molecular structures. The code is publicly available under the GNU Public License (GPLv3) on GitLab.^55^

**FIG. 3.**
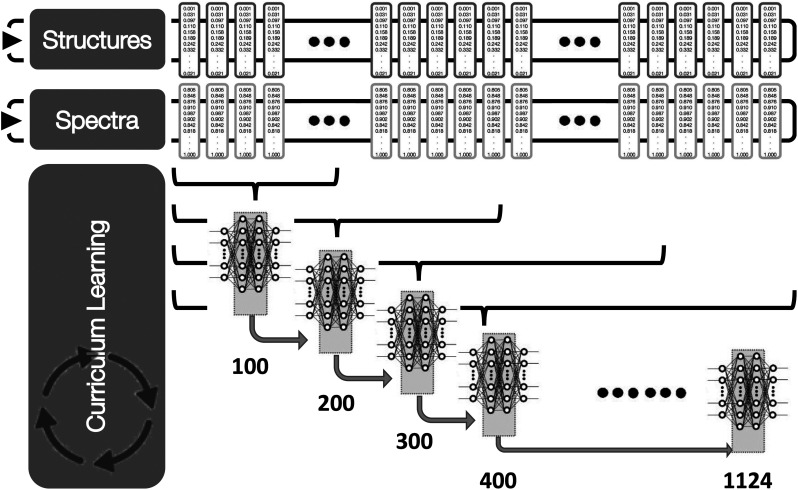
Schematic of the curriculum learning based training adopted in this work. For the latter, 100 spectrum-structure pairs are selected at random and used to train a DNN. Once completed, another 100 spectrum-structure pairs are added at random, with the previous model used a guess for the subsequent training cycle. This is repeated until all the training data are included within the model.

Training of the neural network, shown schematically in Fig. 3 follows an approach inspired by curriculum learning (CL).^56^ CL is a strategy which aims to training a machine learning model from easier data to more complex data, which imitates the meaningful learning order in human curricula. In the present work, the complexity arises from the diversity in the training set. Consequently, we initially select 100 spectrum–structure pairs at random and train the DNN described above. Once completed, another 100 spectrum–structure pairs are added at random to the training set and the previous model used a guess for the subsequent training cycle. This is cycle is repeated until all the training data are included within the model. In contrast to the random sampling, we have also assess furthest-point and closest point sampling,^57^ where by the most (dis)-similar spectra were chosen. We note that during testing this approach, we assessed four different sampling methods, namely,: random sampling, furthest point sampling, closest point sampling and uncertainty-based sampling. Both the furthest and closest point sampling calculates the Euclidean distance between the structural descriptors in the training sets and adds the next 100 based upon the those which are either furthest or closest to the existing samples in the training set. The uncertainty based sampling, estimates the uncertainty of samples not in the training set, using the bootstrapping approach,^29^ it then adds spectra exhibiting either the largest or smallest uncertainty. During testing we found that while each method may slightly differ at small training sets (<500 samples), they all converge to the same performance when all training samples are included. In addition, the method could be sensitive to the initial 100 spectra chosen. As for the sampling method, a small difference can be observed for small training sets (<500 samples), but this difference disappears when all training samples are included.

## III. RESULTS

In the following, we demonstrate the Δ-learning model proposed at the Rh L_3_-edge. Initially, we train the model and demonstrate its performance on a general dataset, before applying it to time-resolved Rh L_3_-edge spectra tracking the C–H activation of octane by a cyclopentadienyl rhodium carbonyl complex.^35^

### A. Performance of the Δ-learning model

**FIG. 4.**
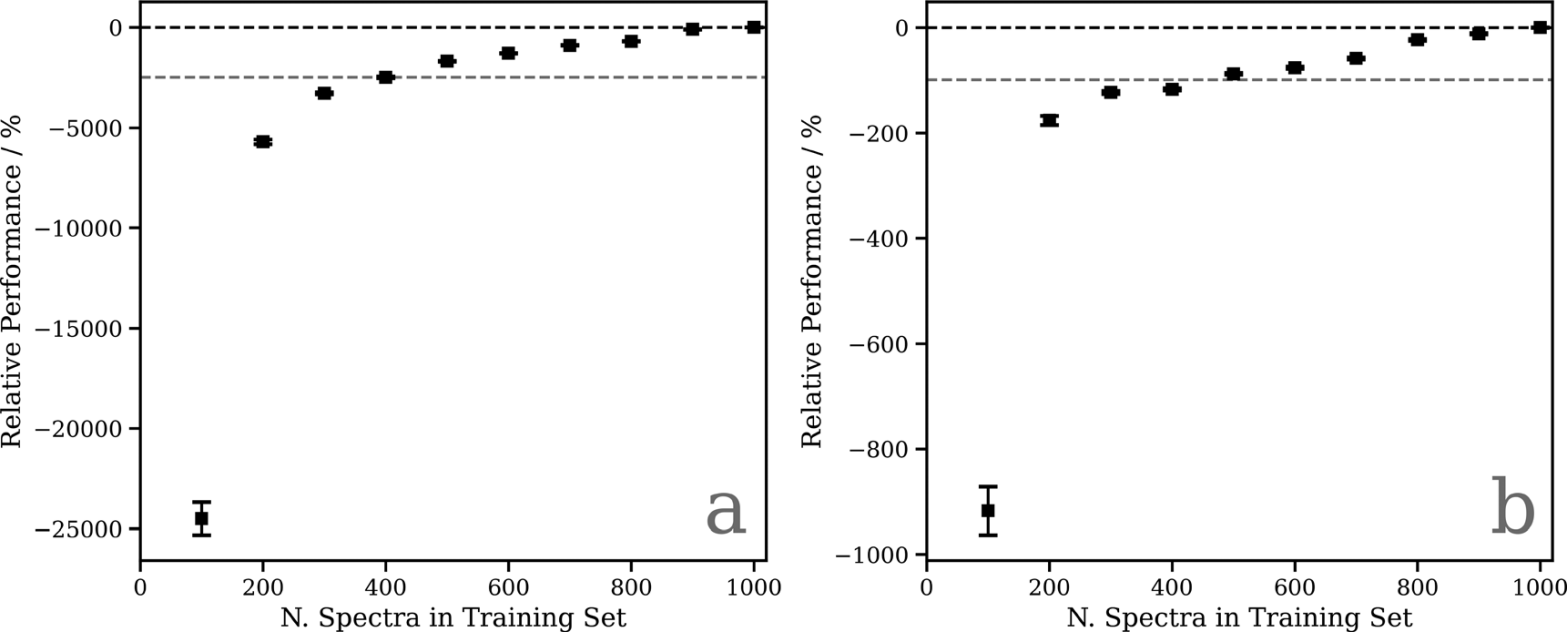
Relative performance of the DNN at the Rh L_3_-edge as a function of the number of training samples. (a) The model trained on the TDDFT(B3LYP) spectra and (b) The model trained on the Δ, i.e*.,* $\mu {\left(E\right)}^{H}-\mu {\left(E\right)}^{L}$. Data points are averaged over 50 K-fold cross-validated evaluations; error bars indicate one standard deviation.

Figure 4 shows the relative performance of our DNN (i.e*.,* the percentage difference between the calculated and predicted spectra relative to the best-performing model for that figure panel) as a function of the number of training samples for the models that directly learn the whole spectra (a) and the Δ-learning model. Both exhibit an initially rapid increase to ∼400 samples, followed by a slower decline. The remaining slow decline indicates that convergence is not entirely achieved and suggests that there is still scope to improve further on the results communicated here by growing/optimizing the dataset. However, the changes are small as chemical space (i.e*.,* the diversity of structures included in the training set compared to the testing set) is well represented and therefore more targeted strategies are required to identify the areas of improvement. The gray dashed line in both figures indicates the performance of the model if CL is not used, and it is clear that this approach gives rise to a substantial improvement in performance for both models.

**FIG. 5.**
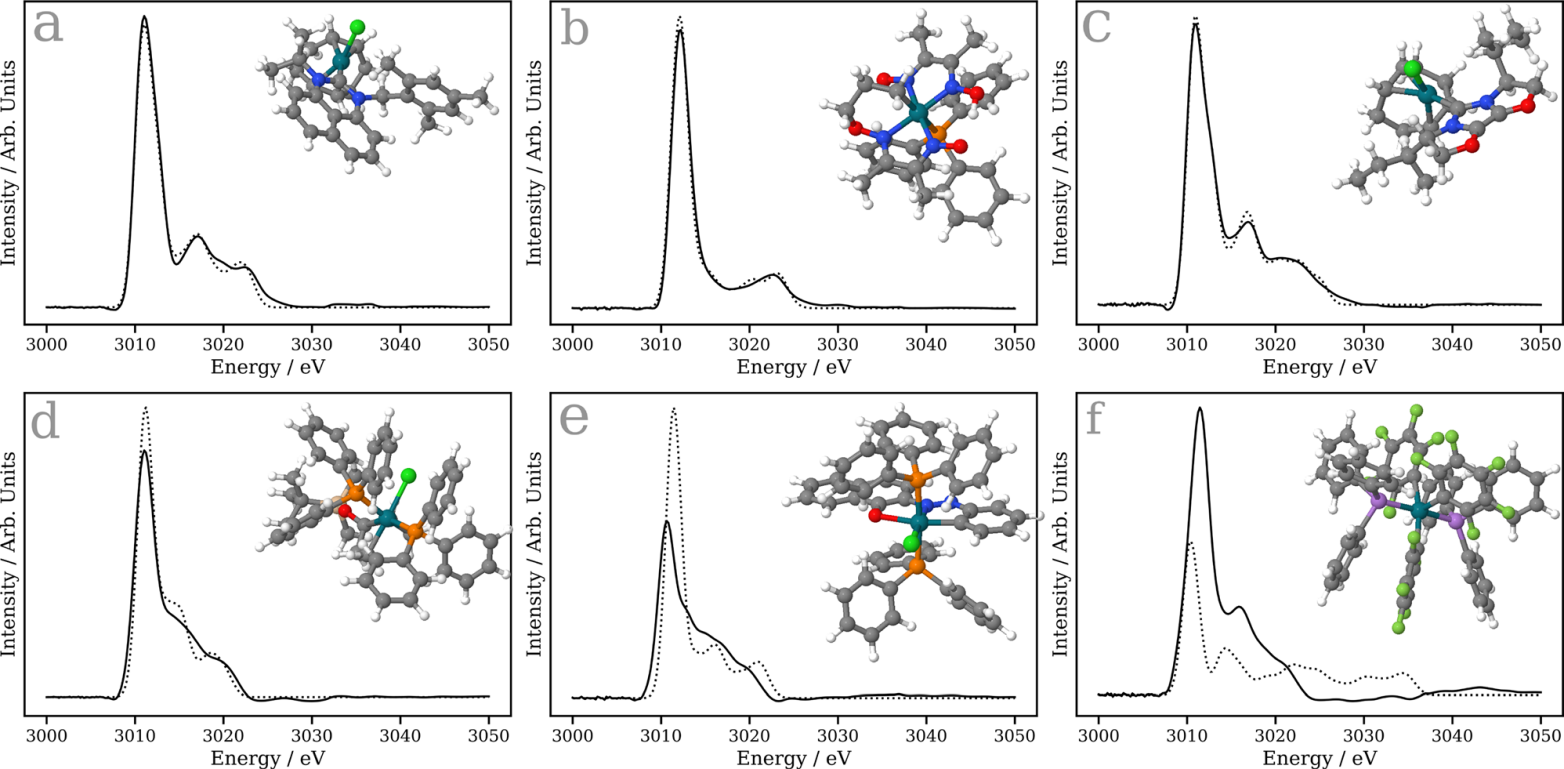
Example Rh L_3_-edge XANES spectra. The upper three panels show spectra from the 0th–10th percentile, i.e., the best performers when held-out set is ranked by MSE. The lower three panels show spectra from the 90th–100th percentile, i.e*.,* the worst performers. The solid black line is the spectrum predicted by the Δ-learning model, while the dashed line is the spectrum calculated using TDDFT(B3LYP). The structures are shown inset and correspond to (a) EFULAU (2.3%) (b) NAXLUT (2.9%) (c) POVQUP (3.2%) (d) AQOFIX (23.3%) (e) FIBJIM (46.6%), and (f) SERTAK (61.3%), where these six-character labels correspond to the Cambridge Structural Database (CSD) codes for the samples. The percentage in bracket refers to the percentage difference between the calculated and predicted spectra.

To assess the performance of the Δ-learning model, we calculate the percentage difference between the calculated spectrum using TDDFT(B3LYP) and the predicted spectrum using the Δ-learning model for 124 held-out examples. The median percentage difference is 5.1%, with the lower and upper quartiles situated at 4.7% and 9.8%, respectively. The tight interquartile range of 5.1% testifies to the balanced performance of the Δ-learning model. To provide context to these percentage differences, Fig. 5 show six example Rh L_3_-edge XANES spectra. The upper three panels show spectra from the 0th–10th percentile, i.e*.,* the best performers when held-out set is ranked by MSE. The lower three panels show spectra from the 90th–100th percentile, i.e*.,* the worst performers. The percentage difference for the upper panels are all <3.2%, comparatively close to the median performance, while the worst performers all exhibit percentage differences >23%, and in these cases the main source of the error is in the intensity of the white line transition. In the case of the worst performers, the poor predictions can be rationalized by the small number of phosphorus, fluorine and arsenic containing molecules in the training set, and therefore this can likely be improved by increasing this in future dataset.

Overall, these results demonstrate the ability of the MLP to operate within a Δ-learning strategy and facilitate accurate predictions of Rh L_3_-edge spectra at TDDFT(B3LYP) level with the computational expense of a TDDFT(BLYP) simulations. The median percentage error for the Δ-learning model is lower than that found for the direct model, using TDDFT(B3LYP) spectra, which is 6.5% and so in Sec. III B we seek to exemplify the performance of the model using simulations of the Rh L_3_-edge spectra tracking the C–H activation of octane by a cyclopentadienyl rhodium carbonyl complex.

### B. Tracking the ligand exchange dynamics of C–H activation

Having developed and assessed the performance of the network in the previous section, we now apply our Δ-learning model to a recent time-resolved x-ray spectroscopic study to track the ligand exchange dynamics of C–H activation.^35^ In this work, the authors demonstrated that changes in oxidation state as well as valence-orbital energies and character, identified using changes in the Rh L_3_-edge spectra, could be used to follow the metal-alkane complex stability and how metal-to-alkane back-donation facilitates C–H bond cleavage by oxidative addition.

**FIG. 6.**
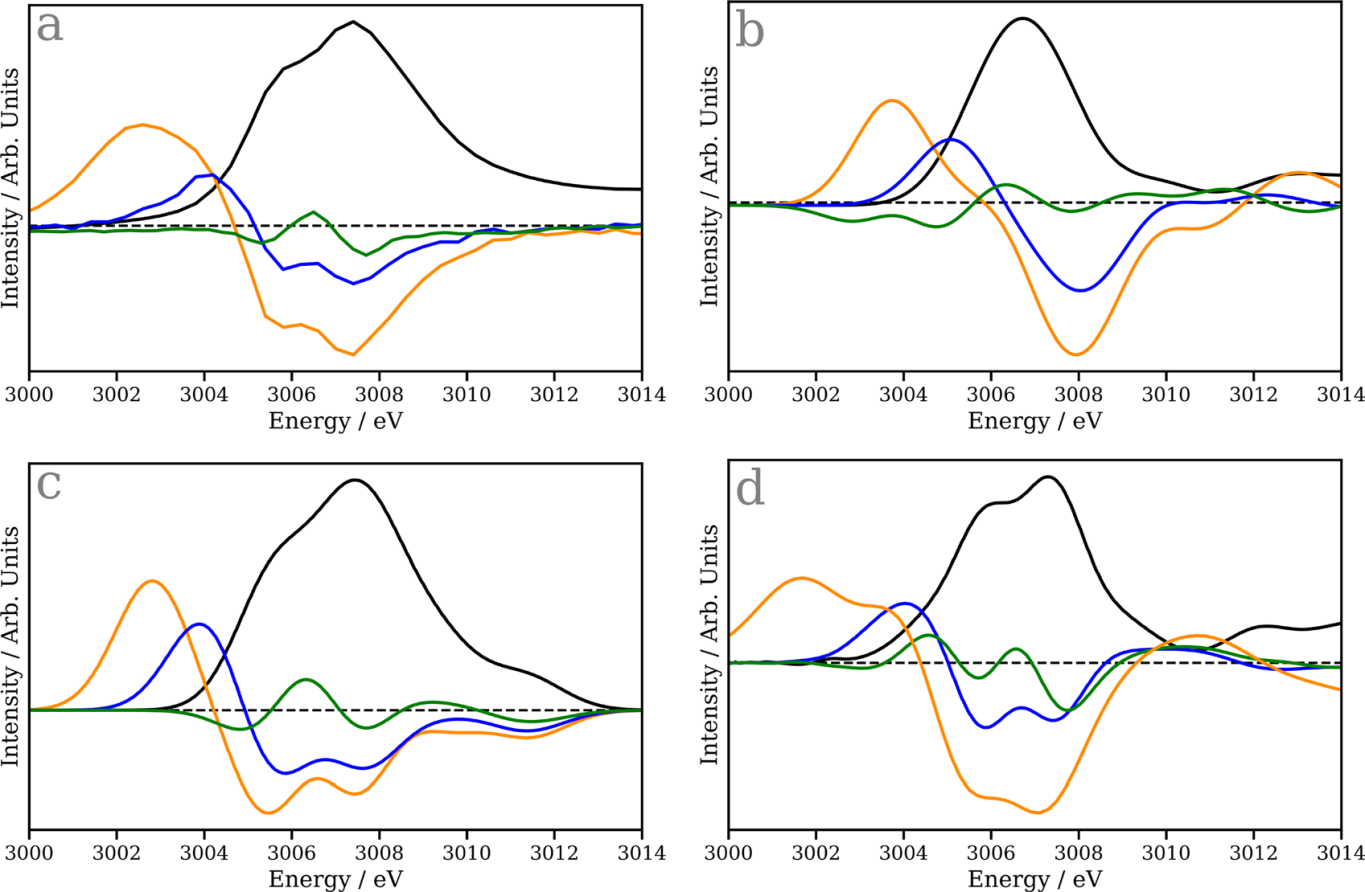
Ground state (black) and transient Rh L_3_ spectra at 250 fs (orange), 10 ps (blue), and >190 ns (green) of cyclopentadienyl rhodium carbonyl, CpRh(CO)_2_. (a) Experimental spectra reproduced from Ref. 35. (b) Spectra calculated using TDDFT(BLYP) (c) TDDFT(B3LYP), and (d) Δ-Learning model. The dashed line represents zero intensity.

The experimental ground state Rh L_3_-edge absorption spectrum of CpRh(CO)_2_ [Fig. 6(a)] shows a main peak at ∼3007.5 eV, with a shoulder at slightly lower energy, ∼3006 eV. This can be interpreted using the TDDFT(B3LYP) calculation, shown in Fig. 6(c) and Ref. 35, which provides good agreement between the experiment and theory. The low energy shoulder, as assigned in Ref. 35, arises from excitation of Rh 2p core electrons into the lowest unoccupied molecular orbital (LUMO) exhibiting Rh 4d character mixed with the C=O ligands. The main band derives from transitions into the LUMO + 1 and LUMO + 2. These exhibit similar Rh 4d mixed with the C=O ligands, but the latter exhibits a substantial Rh 4d and 5s character, which at the L_3_-edge is dipole allowed giving rise to the larger intensity.

In contrast to TDDFT(B3LYP), the TDDFT(BLYP) calculation of the ground state spectrum shown in Fig. 6(b) does not reproduce the two peaks observed in the experiment. While the transitions described above remain present, they occur at the same energy and therefore are indistinguishable. Figure 6(d) shows the spectrum predicted using the Δ-learning model and in agreement with the experiment this provides the double peaked structure, demonstrating that the Δ-learning model is able to overcome the deficiencies of the BLYP calculated spectra and predict a spectrum close to that calculated by TDDFT(B3LYP).

The transient Rh L_3_ spectra at 250 fs (orange) and 10 ps (blue) both exhibit a new transition below the absorption edge. This arises from transitions into the LUMO, whose energy is significantly reduced upon loss of the strong-field C=O. In the present work, seeking to demonstrate the performance of the Δ-learning approach, we have modeled these in these intermediates in their electronic ground state. However, note that in Ref. 35, the authors were not able to unambiguously assign the spectrum to the ground state CpRhCO, and the experimental transient at 250 fs, may also contain components associated with the excited state of CpRh(CO)_2_ and CpRhCO. Therefore, despite the close agreement between experimental and theory in this case, it remains unclear if this state of association of octane occurs in the ground of electronically excited state of CpRhCO.

Upon association of octane (10 ps transition, blue) to form the CpRh(CO)-octane *σ*-complex, the spectrum shifts to slightly higher energy but remains lower than CpRh(CO)_2_. As shown in Fig. 6(d), the Δ-learning model clearly corrects deficiencies in the TDDFT(BLYP) calculations to provide very good agreement between the experiment, TDDFT(B3LYP) and the Δ-learning model. The two exceptions to this are the double peaked structure in the pre-edge feature of the 250 fs (orange) and the >190 ns transient spectrum (green trace). The former is likely associated with the low coordination environment of the Rh complex, which is rare within the present training set and the latter is, as shown In the calculated spectra [Figs. 6(b) and 6(c)], a weak signal and therefore challenges the sensitivity of the model, i.e*.,* if the changes are small, small errors will have a much greater impact than for larger spectral differences. We would expect both to improve upon expansion of the training data.

**FIG. 7.**
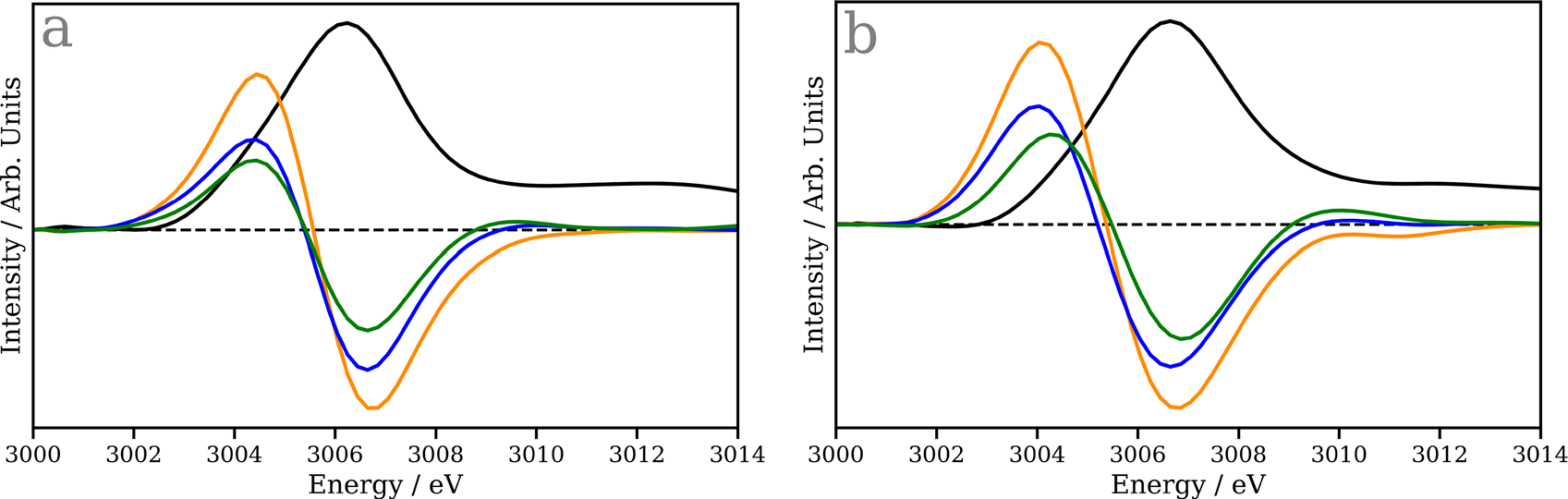
Ground state (black) and transient Rh L_3_ spectra at 250 fs (orange), 10 ps (blue), and >190 ns (green) of cyclopentadienyl rhodium carbonyl, CpRh(CO)_2_. (a) Predictions from a machine learning model trained using TDDFT(BLYP) training set (b) Predictions from a machine learning model trained using TDDFT(B3LYP) training set. The dashed line represents zero intensity.

For comparison, Fig. 7 shows the Rh L_3_-edge XANES spectra predicted from the models trained directly to translate structures into spectra lineshapes trained using the BLYP and B3LYP training spectra i.e*.,* without Δ-ML, as performed in Ref. 16. Both models provide very similar predictions and fail to capture the spectral shape in either the ground state or transient spectra. Indeed the similarity between all of the transient spectra suggests the direct model cannot distinguish between any of the structures during the analysis of the experimental data in Ref. 35 which is likely due to the lack of sensitivity of the model arising from the smaller training dataset.

**FIG. 8.**
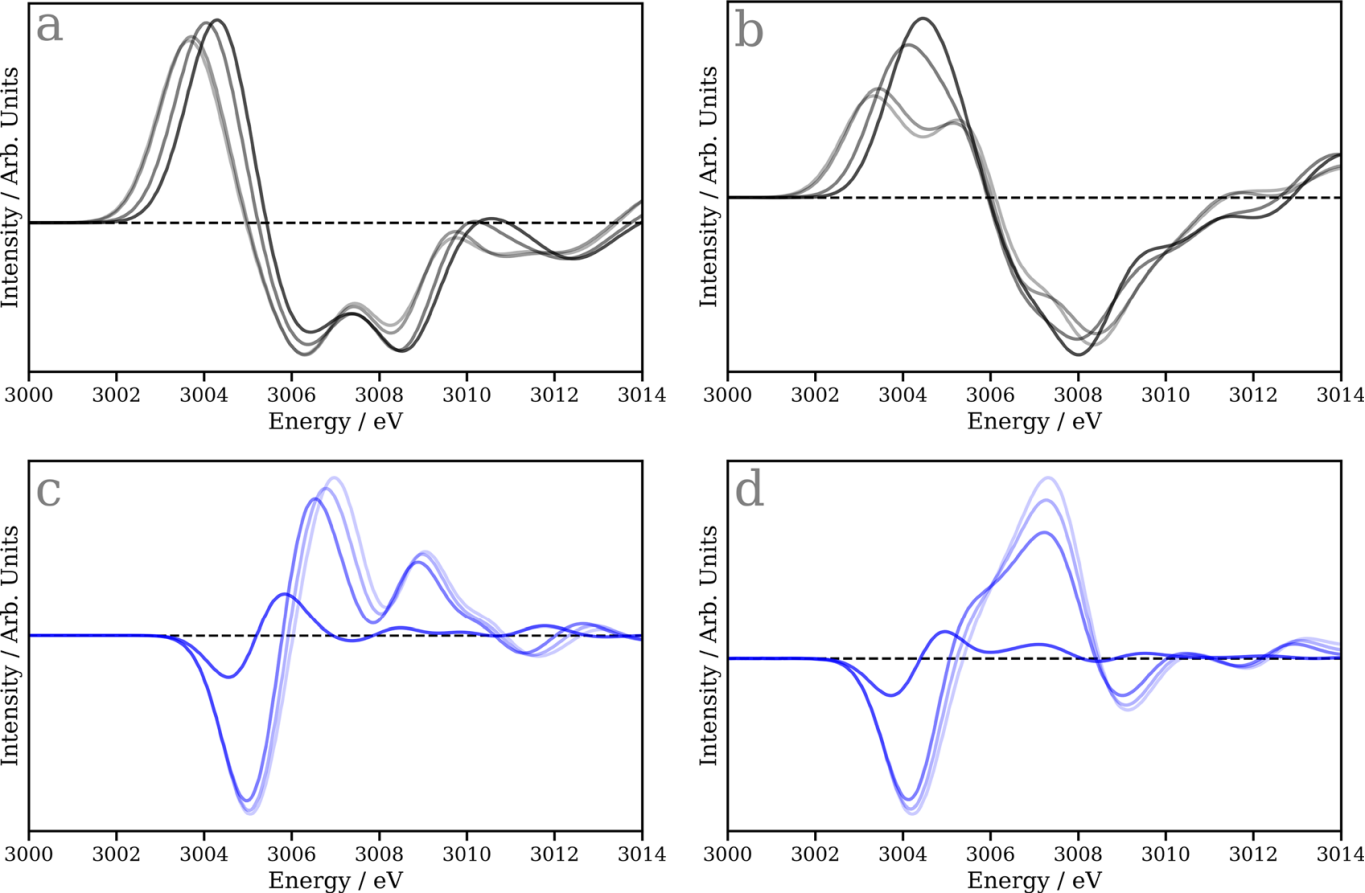
Spectral changes along the two key reaction coordinates: (a) and (b) Dissociation of the CO from CpRh(CO)_2_. The data are plotted as a difference with respect to the starting structure, i.e., CpRh(CO)_2_ ( $R_{{\text{Rh}-C}_{\text{CO}}}$= 1.90 Å). The darker the black line the shorter the Rh-C_CO_ bond length ( $R_{{\text{Rh}-C}_{\text{CO}}}$= 2.75, 3.00, 4.00, and 7.00 Å). The data in panel (a) is calculated using TDDFT(B3LYP), while the data in panel (b) is simulated using the developed Δ-learning model. (c,d) The transformation of CpRh(CO)-octane to CpRh(CO)-H-R. The data are plotted as a difference with respect to the starting structure, i.e*.,* CpRh(CO)-octane ( $R_{Rh-C_{\text{octane}}}$= 2.55 Å). The darkest blue line represents the starting point, i.e*.,* CpRh(CO)-octane, while the lightest blue corresponds to CpRh(CO)-H-R ( $R_{{\text{Rh}-C}_{\text{octane}}}$= 2.35, 2.17, 2.12, and 2.09 Å). The data in panel (c) is calculated using TDDFT(B3LYP), while the data in panel, and (d) is simulated using the developed Δ-learning model.

To illustrative the sensitivity of the Δ-learning model to small structural changes, in contrast to the direct model, Fig. 8 shows the spectral changes (represented as a difference with respect to the starting structure of the reaction coordinate) along the two potential reaction coordinates namely, the dissociation of CO from CpRh(CO)_2_ and the transformation of CpRh(CO)-octane to CpRh(CO)-H-R. Figures 8(a) and 8(b) show the dissociation of CO from CpRh(CO)_2_, with Fig. 8(a) being the spectra calculated using TDDFT(B3LYP), while Fig. 8(b) is predicted using our Δ-learning model. Overall, there is good agreement between the two with the derivative profile consistent with the generation of a pre-edge peak and it shifting to lower energies during dissociation, proceeds. The Δ-learning model exhibits a double peak in the pre-edge, but consistent with TDDFT(B3LYP), the main band loses intensity and shifts to lower energy. Above 3006 eV in the region of the white line, the Δ-learning reproduces the general double peaked shape observed in the spectra calculated using TDDFT(B3LYP), but these are slightly too close together. In comparison to the changes observed below 3006 eV, this region of the spectrum exhibits much smaller changes which is consistently reproduced between both models.

Figures 8(c) and 8(d) show the spectral changes associated with the transformation of CpRh(CO)-octane to CpRh(CO)-H-R, with Fig. 8(c) being the spectra calculated using TDDFT(B3LYP) and Fig. 8(d) being predicted using our Δ-learning model. The first difference (the darkest blue line) shows excellent agreement between the TDDFT(B3LYP) calculated and Δ-learning predicted spectra. For spectral changes close to the CpRh(CO)-H-R structure (lighter blue lines) clear deviations begin to emerge. The TDDFT(B3LYP) calculated difference shows two principle positive features at 3007 and 3009 eV, which both increase in intensity and shift to higher energies closer to the CpRh(CO)-H-R structure. The Δ-learning predicted spectra also shows two main features, which both shift to higher energies, however their intensities are the wrong way round, which is expected as the difference spectrum associated with CpRh(CO)-H-R structure is the poorest agreement with experiment shown in Fig. 6.

## IV. DISCUSSION AND CONCLUSION

In this article, we have introduced a Δ-learning strategy aimed at transforming spectral lineshapes from a low-level of theory to a higher-level of theory. This composite approach has the benefit of combining fast calculations with a simple correction scheme based upon our machine learning model which can achieve predictions comparable to higher levels of theory, without the additional computational expense. We have applied the developed models to time-resolved Rh L_3_-edge spectra tracking the C–H activation of octane by a cyclopentadienyl rhodium carbonyl complex^35^ and demonstrated the effectiveness of the Δ-learning approach for translating the TDDFT(BLYP) spectroscopic observables to those of the TDDFT(B3LYP) level.

The proof-of-concept Δ-learning work has demonstrated that one can reach the accuracy of a higher-level quantum chemistry core-hole spectrum at lower computational burden. Future work should focus on extending this, especially in term of the size of the training set and the Δ, i.e*.,* the difference in quality of the low and high level quantum chemistry methods used. For the latter, a more significant computational advantage could be obtained using the difference between a quasi-one-electron approach based upon Kohn–Sham orbitals^58^ and the restricted open-shell configuration interaction (ROCIS) method,^59^ the latter of which has shown to be highly effective for simulating L_3_-edge,^60^ without the requirement for highly bespoke system specific inputs associated with the restricted active space methods.^11^ The larger expected size of the Δ in this case is likely to require a larger and more diverse training set, which will be the focus of future work.

## Data Availability

The data that support the findings of this study are openly available in GitLab at gitlab.com/team-xnet/xanesnet-keras, Ref. 55 and GitLab at gitlab.com/team-xnet/training-sets, Ref. 61.
